# Oxidative Desulfurization of Petroleum Distillate Fractions Using Manganese Dioxide Supported on Magnetic Reduced Graphene Oxide as Catalyst

**DOI:** 10.3390/nano11010203

**Published:** 2021-01-14

**Authors:** Waqas Ahmad, Atiq Ur Rahman, Imtiaz Ahmad, Muhammad Yaseen, Badrul Mohamed Jan, Minas M. Stylianakis, George Kenanakis, Rabia Ikram

**Affiliations:** 1 Institute of Chemical Sciences, University of Peshawar, Khyber Pukhtunkhwa 25120, Pakistan; atiqurrahman329@gmail.com (A.U.R.); dr_imtiaz@uop.edu.pk (I.A.); myyousafzai@uop.edu.pk (M.Y.); 2 Department of Chemical Engineering, University of Malaya, Kuala Lumpur 50603, Malaysia; badrules@um.edu.my; 3 Institute of Electronic Structure and Laser, Foundation for Research and Technology-Hellas, N. Plastira 100, Vasilika Vouton, GR-700 13 Heraklion, Greece; stylianakis@iesl.forth.gr (M.M.S.); gkenanak@iesl.forth.gr (G.K.)

**Keywords:** metal oxides catalyst, GO support, desulfurization, reduction of GO, H_2_O_2_/HCOOH

## Abstract

In this study, oxidative desulfurization (ODS) of modeled and real oil samples was investigated using manganese-dioxide-supported, magnetic-reduced graphene oxide nanocomposite (MnO_2_/MrGO) as a catalyst in the presence of an H_2_O_2_/HCOOH oxidation system. MnO_2_/MrGO composite was synthesized and characterized by scanning electron microscope (SEM), energy dispersive X-ray spectroscopy (EDX), Fourier transform infrared spectroscopy (FTIR), and X-ray diffraction (XRD) analyses. The optimal conditions for maximum removal of dibenzothiophene (DBT) from modeled oil samples were found to be efficient at 40 °C temperature, 60 min reaction time, 0.08 g catalyst dose/10 mL, and 2 mL of H_2_O_2_/formic acid, under which MnO_2_/MrGO exhibited intense desulfurization activity of up to 80%. Under the same set of conditions, the removal of only 41% DBT was observed in the presence of graphene oxide (GO) as the catalyst, which clearly indicated the advantage of MrGO in the composite catalyst. Under optimized conditions, sulfur removal in real oil samples, including diesel oil, gasoline, and kerosene, was found to be 67.8%, 59.5%, and 51.9%, respectively. The present approach is credited to cost-effectiveness, environmental benignity, and ease of preparation, envisioning great prospects for desulfurization of fuel oils on a commercial level.

## 1. Introduction

Oxidative desulfurization (ODS) is currently enjoying popularity as a highly efficient and conveniently operating alternative or complementary to hydrodesulfurization (HDS) technique for resilient sulfur compounds removal from petroleum distillate fractions used as transportation fuels [1,2]. The process is performed at ambient operating conditions in two steps, i.e., oxidation of sulfur-containing compounds followed by extraction via a polar solvent [3]. ODS converts sulfur-containing compounds into highly polar sulfones, which can be easily separated by a polar solvent like acetic acid, dimethyl sulfoxide, and methanol [4]. ODS process offers manifold advantages over the conventional HDS one, which can be operated at liquid phase under mild operating conditions, i.e., close to ambient. In this context, the use of hydrogen gas, a specialized high-pressure and high-temperature reactor, as well as high efficiency and selectivity for alkylated dibenzothiophene removal, are not considered as requirements [5]. Due to the electron-donating character of alkyl groups in alkylated dibenzothiophenes, the high electron density in a sulfur atom increases its ease of oxidation, and therefore, alkylated dibenzothiophenes are conveniently treated during the ODS process, unlike in the case of HDS [6].

In the ODS process, the selective oxidation of sulfur compounds is achieved by various types of oxidants, such as H_2_O_2_, molecular oxygen, hydrogen peroxide, formic acid, tert-butyl hydroperoxide, ozone, perchlorate, hypochlorite, potassium permanganate, and nitrogen oxide [7,8,9]. Since ODS efficiency can be further enhanced by using a variety of catalysts, different types of catalyst-oxidant systems like NaClO/Mn–Co–Mo/Al_2_O_3_ [10], H_2_O_2_/Mo/γ–Al_2_O_3_ [11], H_2_O_2_/WOx/ZrO_2_ [12], H_2_O_2_/V_2_O_5_ [4], H_2_O_2_/activated carbon TBHP/(Me_3_TACN)Mn [13], TBHP/Bi–Mo/Siral [14], TBHP/Ti–MCM-41 and MMO/GO [15], and various types of polyoxometalate [16,17] have been studied. Each of these catalytic ODS systems follows different oxidation mechanisms of sulfur compounds [18]. Sulfur removal has also been investigated by photooxidation using various photocatalysts [19,20]. 

In general, metal oxide catalysts lead to the formation of peroxo-complexes, which further oxidizes dibenzothiophene (DBT) in the non-polar phase. Commonly used metal oxides for ODS of modeled and real oil samples include MoO_2,_ MnO_2_, PdO, TiO_2_, CoO, etc. [21,22,23]. In order to enhance their catalytic activity and increased surface area, metal oxides are loaded on various supports. For this purpose, studies have been reported in the literature, including supporting materials such as montmorillonite (MMT), TiO_2_, Al_2_O_3_, and graphene oxide (GO) [24]. The utilization of graphene-related materials as support frameworks for functional nanoparticles has emerged as a promising research area, utilizing a combination of properties such as high specific surface area, lightweight, chemical inertness, mechanical robustness, and excellent electrical and thermal conductivity [25]. Many studies have shown that when using graphene derivatives as supports, the catalyst’s activity is manifoldly enhanced due to their unique mechanical, morphological, and electrical properties, in addition to their high specific surface area [26]. Other studies have demonstrated that GO exhibits high compatibility with oxidative adsorptive desulfurization systems [27], while reduced GO (rGO) can also enhance the activation of oxidants during the ODS process [28]. Likewise, due to the matching of 2D geometry and charge complementarity, rGO is expected to be an excellent support for metal oxide [15]. 

Hence, in the current study, ODS of DBT was performed using a composite of manganese dioxide-supported and magnetic-reduced graphene oxide (MnO_2_/MrGO) in the presence of an HCOOH/H_2_O_2_ oxidation system under ambient operating conditions. The composite catalyst was characterized by scanning electron microscope (SEM), Fourier-transform infrared spectroscopy (FTIR), energy dispersive X-ray (EDX), and X-ray diffraction (XRD) techniques. DBT conversion was investigated under various reaction conditions like different reaction times and temperatures, oxidant and catalyst dose, etc. Finally, the activity of the catalyst was also tested for ODS of real gasoline, diesel, and kerosene samples under the optimized conditions set. 

## 2. Experimental

### 2.1. Chemicals and Reagents 

All chemicals and reagents used were of analytical grade. Graphite powder, potassium permanganate (KMnO_4_) 99%, sulfuric acid (H_2_SO_4_) 98%, hydrogen peroxide (H_2_O_2_) 30% (*v*/*v*), iron (III) chloride hexahydrate (FeCl_3_·6H_2_O) 98%, iron (II) sulfate heptahydrate (FeSO_4_·7H_2_O) 99%, ammonia (NH_4_OH) 25%–28%, diamine hydrate (H_2_NNH_2_·H_2_O) 80%, manganese sulfate monohydrate (MnSO_4_·H_2_O) 99%, potassium hydroxide (KOH) 85%, formic acid (HCOOH) 95%, and n-heptane 99% were purchased from Sigma-Aldrich, St. Louis, MO, USA. Methanol (CH_3_OH) 99% was provided by Fisher Scientific, Pittsburgh, PA, USA. Gasoline (RON 92), kerosene, and diesel oil were collected from a Shell oil filling station in Peshawar City, Pakistan.

### 2.2. Preparation of MnO_2_/MrGO Composite Catalyst 

Graphene oxide was synthesized from commercial graphite powder by a modified Hummers method [22]. More specifically, powder graphite (2 g) was dispersed in a mixture of H_2_SO_4_ (92 mL) and NaNO_3_ (1 g). The mixture was cooled down to less than 10 °C, over an ice bath. KMnO_4_ (12 g) was added to the mixture, stirred for 30 min, and then allowed to settle down overnight. Deionized water (184 mL) was added to the suspension and heated up to 95 °C for 15 min; after this, H_2_O_2_ was added dropwise till the color of the suspension changed to yellow, which indicated the formation of graphene oxide in the suspension. 

For the reduction of graphene oxide, the mixture was refluxed at 90 °C for 4 h in the presence of diamine hydrate as a reducing agent. A black precipitate of reduced graphene oxide (rGO) was then produced. rGO was collected by filtration, washed thoroughly with deionized water, and finally, dried under vacuum.

Reduced graphene oxide was magnetized through in-situ magnetization [29] to prepare magnetic-reduced graphene oxide (MrGO). To this end, about 0.1 g of rGO was dispersed in 50 mL of distilled water and then added to an aqueous solution of FeCl_3_.6H_2_O and FeSO_4_.7H_2_O (2:1) at 90 °C. The mixture was turned alkaline by the addition of 15 mL of ammonia solution, and then H_2_O_2_ was added under vigorous stirring until the black precipitate of magnetic reduced graphene oxide was formed, which was collected, washed, and dried.

MnO_2_/MrGO nanocomposite was synthesized by dispersing MrGO in a solution of manganese sulfate monohydrate (MnSO_4_·H_2_O) at 80 °C and a mixture of KMnO_4_ (0.16 g) and KOH (0.23 g), dissolved in deionized water (5 mL), followed by rapid addition to the mixture. The mixture was refluxed for 1.5 h, and a black precipitate of 1.69 g of MrGO-MnO_2_ nanocomposite was formed, which was separated by an external magnet, washed, and dried in an oven under vacuum. About 1 g dry mass was recovered and stored in vials. 

### 2.3. Characterization of the Catalyst

The catalyst MnO_2_/MrGO, synthesized in the lab, was characterized by SEM, EDX and XRD, and FTIR analyses. SEM and EDX analyses were carried out using a SEM (Model JEOL-Jsm-5910; Tokyo, Japan), while XRD patterns were conducted using X-ray diffractometer (XRD; model JDX-9C, JOEL, Tokyo, Japan) with CuKα radiation (1.54178 A° wavelength) and a nickel filter. FTIR analysis of the catalyst was carried out by an FTIR spectrophotometer (Schimadzu-A60, Kyoto, Japan). 

### 2.4. ODS Activity of the Catalyst 

ODS of the modeled oil sample (0.03 g DBT in 15 mL n-heptane) with a concentration of 1200 ppm DBT was conducted with ultrasonication in the presence of MnO_2_/MrGO catalyst and H_2_O_2_-HCOOH oxidation system. In a typical experiment, DBT in solution (15 mL), the catalyst (0.06 g), and H_2_O_2_ and HCOOH (2 mL) in 1:1 were taken in a two-neck flask fitted with a reflux condenser. The flask was put in an ultrasonic bath and sonicated for 1 h, under atmospheric pressure at ambient temperature (about 27 °C). After oxidation, the catalyst was collected by the external magnetic field, and the sample was subjected to analysis for change in DBT concentration (ppm). 

Catalytic ODS experiments were further carried out under different conditions of temperature, reaction time, catalyst dose, and oxidant concentration, in order to optimize the process parameters. The catalytic ODS of the real oil samples was studied under optimized conditions, using the same procedure as adopted for the modeled oil sample. 

### 2.5. Analyses

The concentration of DBT in the modeled oil sample was analyzed by HPLC (Sykam, Eresing, Germany), equipped with BDS Hypersil C18 column (dim. 250 × 4.6 mm) and a UV detector. Methanol was used as the mobile phase; analysis was carried out at λ_max_ of 320 nm. % DBT conversion was calculated using Equation (1),

(1) $$DBT\;conversion\;\left(\%\right)=\frac{\;C_{o}-C}{C_{o}}\;\times \;100$$

where *C*_o_ and *C* represent the initial and post ODS concentrations of DBT, respectively. 

Total sulfur in gasoline, kerosene, and diesel oil was determined by a CHNS elemental analyzer (EL-III, Hanau, Germany) provided with an auto-sampler and a TCD detector. The percentage of desulfurization in real oil samples was calculated using the relation as given above (Equation (1)). 

## 3. Results and Discussion 

### 3.1. Characterization of the Catalyst 

**Figure 1:**
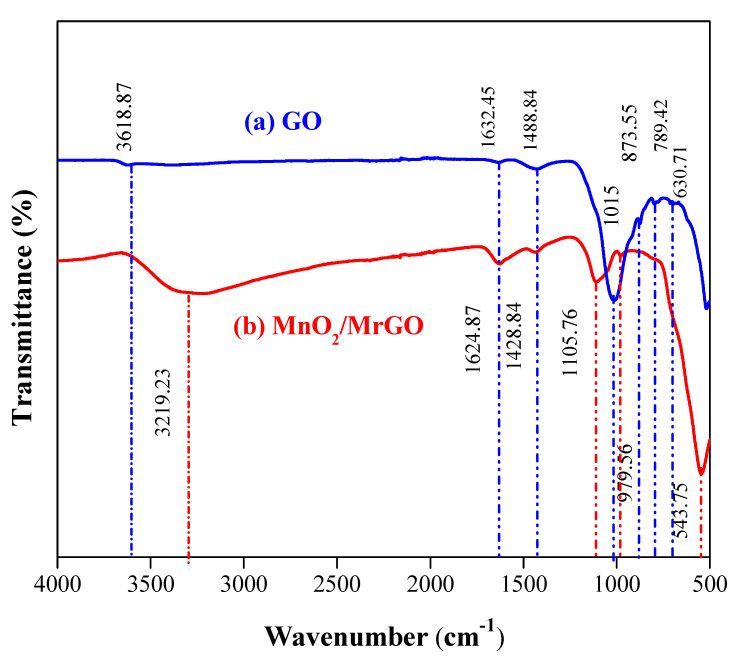
Fourier-transform infrared spectroscopy (FTIR) spectra of (**a**) graphene oxide (GO) and (**b**) MnO_2_/MrGO.

FTIR spectra of GO and MnO_2_/MrGO are displayed in Figure 1. The spectrum of GO shows prominent peaks at 3618.87 cm^−1^, which corresponds to O–H stretching vibrations of carboxylic acids [30]. Characteristic absorption peaks appearing at 1428.84, 1624.18, 1007.33, and 1488.84 cm^−1^ in the FTIR spectra of pure GO showed the presence of carboxyl O=C=O, C=O aromatic, C=C, alkoxy C–O stretching vibrations, and epoxy C–O, respectively [30]. Furthermore, the FTIR absorption band of pure GO at 3618.87 cm^−1^, displayed in Figure 1a, is due to the presence of OH of the absorbed molecules of water. Compared with Figure 1b, it shows an additional sharp peak at around 552.16 cm^−1^ which is attributed to Fe–O vibrations of Fe_3_O_4_ [31,32]. Another sharp peak at 630.71 cm^−1^ appears in Figure 1 for the Fe_3_O_4_–rGO–MnO_2_ sample, which is absent in the other spectra. This fact is attributed to some interactions between iron and manganese atoms [30,33]. 

**Figure 2:**
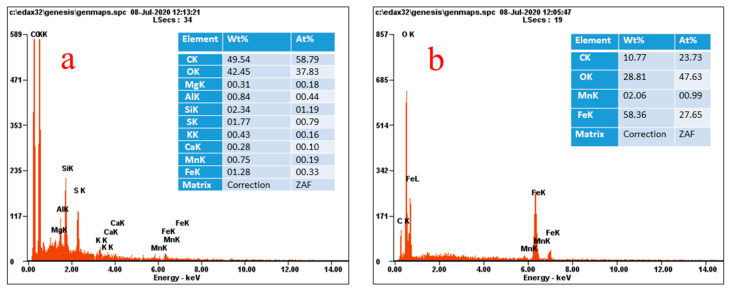
Energy dispersive X-ray (EDX) spectra of (**a**) GO and (**b**) MnO_2_/MrGO_._

The mineralogical composition of the catalyst was studied by EDX; the EDX profiles of GO and MnO_2_/MrGO composite are given in Figure 2a,b. From the data, it is clear that although GO consists of C and O as its major elements (59% and 38%, respectively), trace quantities of Si, S, Mn, and Fe are also indicated with a total amount of less than 1%, which may be due to impurities in the commercial graphite. In the case of MnO_2_/MrGO, the major elements present were C (10%), O (28%), Fe (58%), and Mn (2%). The presence of Mn clearly confirms the successful synthesis of MnO_2_/MrGO composite. Moreover, the data show that the percentage weight of Mn in MnO_2_/MrGO is close to its theoretical weight% loaded in MrGO. 

**Figure 3:**
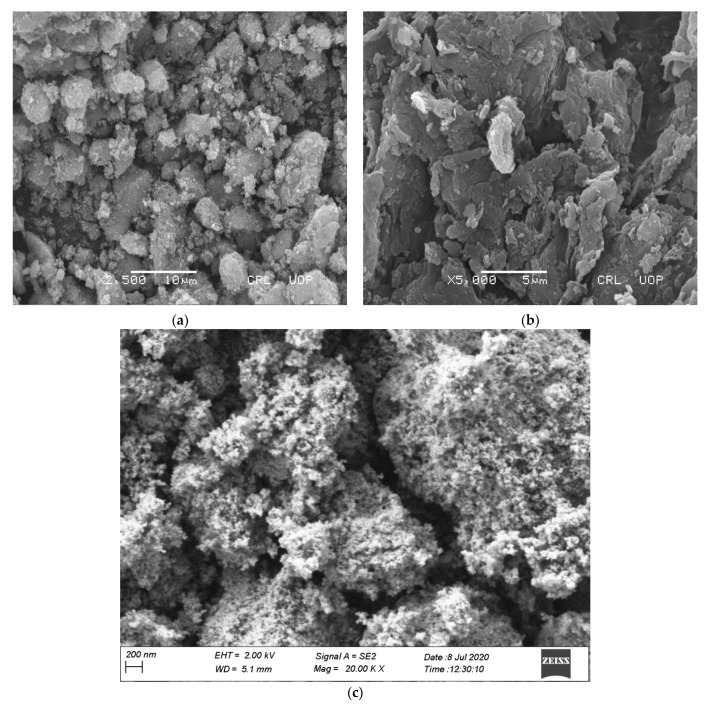
Scanning electron microscope (SEM) images of (**a**) GO, (**b**) MnO_2_/MrGO, and (**c**) field emission scanning electron microscope (FESEM) image of MnO_2_/MrGO_._

The SEM micrographs of GO and MnO_2_/MrGO, are presented in Figure 3a,b. The micrograph of GO shows a rough, layered, and wrinkled morphology, while major fissures and caves are visible. The texture resembles layered sheets like the two-dimensional structure of graphene oxide [34]. The void spaces between the layer and edge can be also seen in the image, suggesting that the material can offer a high surface area for the reaction, while some bulk aggregates on the surface of GO in Figure 3b correspond to the proper agglomeration of catalyst. More interestingly, the surface of MnO_2_/MrGO differs compared with that of pure GO, as shown in Figure 3b. It shows a uniform spreading of MnO_2_ particles on the MrGO matrix, illustrating their successful composition. On the other hand, the micrograph of MnO_2_/MrGO shows an irregular, non-uniform particle size distribution. Figure 3c shows the field emission scanning electron microscope (FESEM) image of MnO_2_/MrGO with up to 200 nm magnification, which clearly reveals that magnetic iron oxide and MnO_2_ nanoparticles resemble fine granular particles with the particle size below 50 nm, which are dispersed on graphene flakes. The size of the graphene flakes is non-uniform, but some are nearly 400–800 nm in diameter.

**Figure 4:**
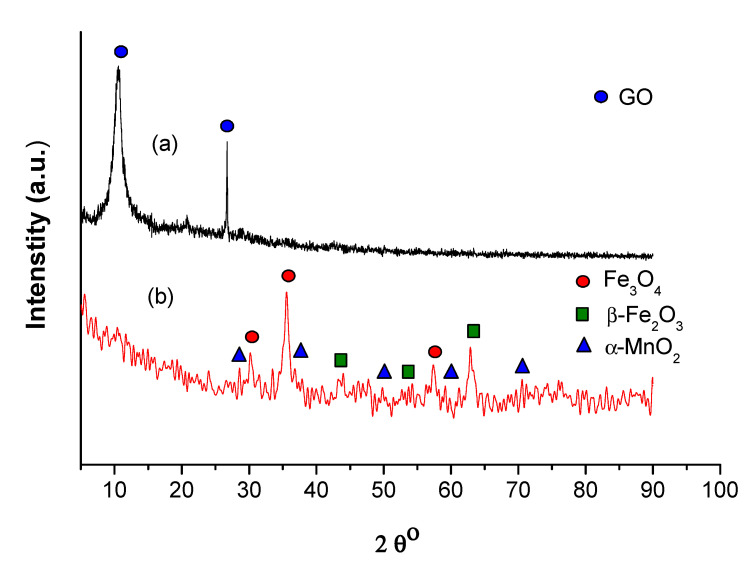
X-ray diffraction patterns (XRD) of (**a**) GO and (**b**) MnO_2_/MrGO_._

The crystallinity and composition of GO and MnO_2_/MrGO composite were investigated by XRD analysis, as shown in Figure 4a,b. The XRD pattern of GO (Figure 4a) shows sharp peaks, indicating better crystallinity. The characteristic GO peak appears at 10.5° 2θ, confirming its successful preparation [30]. A sharp peak at 26.7° corresponds to the existence of some unoxidized graphite fractions in the sample [35]. In the case of MnO_2_/MrGO, the sharp peaks centered at 30°, 35°, and 57° correspond to crystalline patterns of magnetite (α-Fe_3_O_4_) and peaks positioned at 43°, 53°, and 63° indicate maghemite (β-Fe_2_O_3_), which agree with the corresponding standard JCPDS card No. 75-0033 and card No. 39-1346, respectively [36]. These confirm the presence of magnetic iron oxides loaded on the surface of GO. Several low intense peaks positioned at 28.6, 37.5, 49.8, 60.1, and 69.1° are attributed to the characteristic crystalline patterns of manganese dioxide (α-MnO_2_) which match with its reference card No.01-072-1982 [37]. The low intensity of these peaks may be due to the small concentration of MnO_2_ in the composite catalyst. 

### 3.2. ODS Activity of MnO_2_/MrGO

ODS activity of MnO_2_/MrGO composite catalyst was investigated using a modeled oil sample, while the catalyst’s activity was determined in terms of the percentage conversion of DBT. DBT removal was investigated under different conditions of time, initial concentration, temperature, catalyst dose, oxidant dose, and catalyst recycling. 

**Figure 5:**
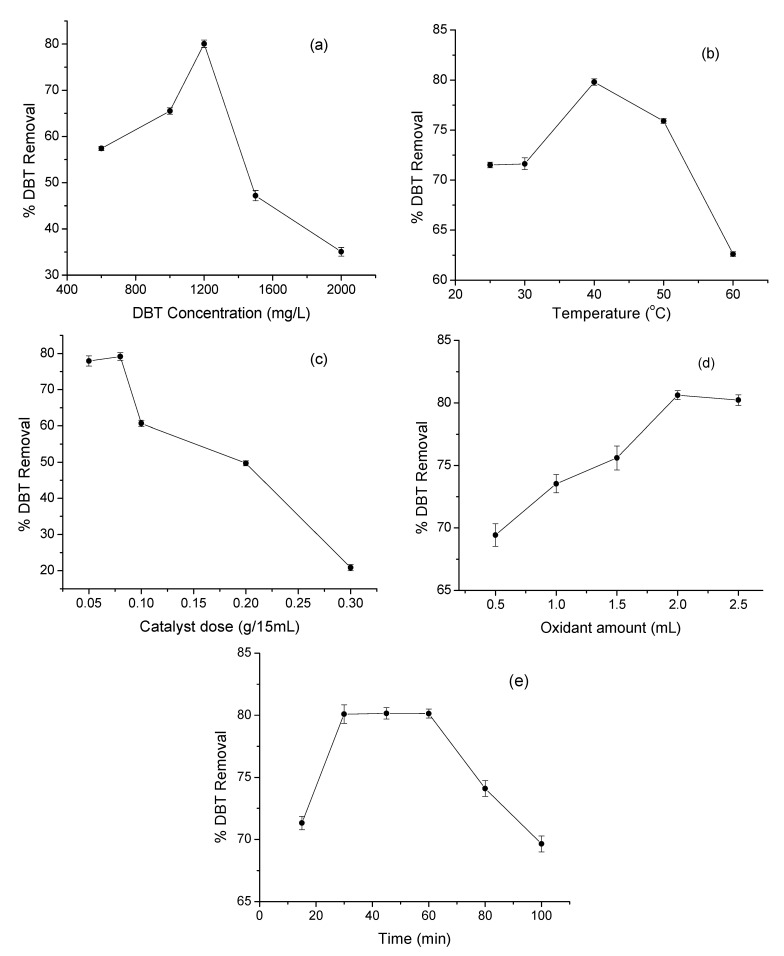
Effect of (**a**) dibenzothiophene (DBT) initial concentration, (**b**) temperature, (**c**) catalyst dose, (**d**) amount of oxidant, and (**e**) time on % DBT removal in the presence of MnO_2_/MrGO catalyst.

It was shown that the catalyst’s activity is strongly affected by DBT concentration because of the interaction between the reactive active sites of the catalyst and the reactant [38]. DBT oxidation was conducted at different initial concentrations ranging from 600 to 2000 ppm, while other experimental conditions, i.e., catalyst dose (0.08 g), reaction temperature (40 °C), reaction time (1 h), and oxidant ratio were kept constant. The results are shown in Figure 5a, indicating that when the initial concentration of DBT was raised from 600 ppm to 1200 ppm, DBT removal linearly increased from 57.45% to 80.11%, albeit with a further increase of the initial DBT concentration, the efficiency declined. It should be mentioned that because the fixed catalyst amount bears a limited number of active sites, which can only carry a proportional number of DBT molecules, over a certain concentration, i.e., 1200 ppm, the number of active sites are insufficient for DBT oxidation [39]. On this basis, the modeled oil sample with 1200 ppm DBT concentration was employed for further study. 

DBT conversion was investigated as a function of temperature in the range of 25 °C to 60 °C, keeping other parameters constant. The extracted results are displayed in Figure 5b, which exhibit that maximum DBT conversion of up to 80% is achieved at 40 °C, whereas above and below 40 °C, DBT conversion declines. It is proved that higher temperatures lead to side reactions and cause rapid dissociation of H_2_O_2_, which leaves DBT unoxidized [40], leading to a lower DBT conversion degree. The majority of literature studies report on the optimum temperature of catalytic ODS in the range of 60 °C to 70 °C. Likewise, the obtained high DBT conversion of 80% at 40 °C demonstrates superior efficiency of the current catalyst-oxidation system. The increase of DBT removal from the modeled oil sample with increasing temperature and reaction time is in agreement with the literature [41].

DBT conversion under different catalyst doses ranging from 0.05 g/15 mL to 0.3 g/15 mL of the modeled oil sample is shown in Figure 5c. The results indicate that maximum DBT conversion of 79% is attained at the catalyst dose of 0.08 g/15 mL, although with further increase in the catalyst dose, a decline in DBT conversion is observed. Although a high catalyst dose provides more active sites for interaction with DBT, leading to higher desulfurization efficiency [42], in the current study, the high activity of the catalyst and the strong oxidizing power of oxidant seem to favor high DBT removal at very low catalyst dosage. The catalyst dose above the optimum level may lead to agglomeration, which blocks the active sites, hence causing a decrease in DBT conversion activity.

In the ODS process, the amount of oxidant consumed directly affects the efficiency and the processing cost [2]. Formic acid and H_2_O_2_ have been used as oxidants for ODS of modeled or real oil samples in the presence of various catalysts during the process when H_2_O_2_ reacts with HCOOH to produce performic acid, which further oxidizes DBT to sulfones [43]. The effect of different concentrations of H_2_O_2_ and formic acid was tested in the presence of MnO_2_/MrGO catalyst by using different volumes, i.e., 0.5, 1.0, 1.5, 2.0, and 2.5 mL of both species. In Figure 5d, it is proved that for both H_2_O_2_ and formic acid, the maximum sulfur conversion of up to 80% is attained when the volume is 2 mL. It has been reported that using an excess amount of aqueous oxidant is helpful for DBT conversion and assists in the extraction of the oxidized product [44]. Apart from the reduction potential, S in DBT acts as a soft base according to the Pearson theory, which would prefer to react with a soft acid [45,46]. 

The effect of reaction time on the catalytic ODS of the modeled oil sample is demonstrated in Figure 5e. It is obvious that DBT conversion rises from 71% to 80% when the reaction time increases from 15 min to 30 min but remains constant till 60 min and then decreases onward from 80% to 69% when the time increases. This could be explained by describing the interaction of oxidizing agents as time proceeds. The reaction of HCOOH and H_2_O_2_ with DBT to produce sulfones needs enough time to be completed and promoted as time increases. However, beyond the optimum time, i.e., 15 min, the drop in sulfur removal degree may be ascribed to the oxidant decomposition, particularly of H_2_O_2_, which is no longer available for oxidation. Hence, the exclusive loss of all oxidants, the equilibrium change, and the oxidation power of reaction media decreases which result a decline in DBT conversion as time progresses [47].

### 3.3. Effect of Temperature and Kinetic Study

**Figure 6:**
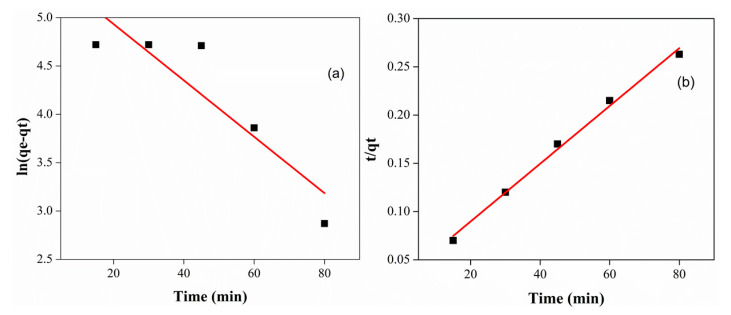
(**a**) Pseudo-first-order and (**b**) pseudo-second-order kinetic models for catalytic oxidative desulfurization (ODS) of DBT in the presence of MnO_2_/MrGO catalysts.

The kinetics of oxidative conversion of DBT in the modeled oil sample in the presence of MnO_2_/MrGO catalysts was studied by applying pseudo-first-order and pseudo-second-order kinetic models to DBT conversion data. The pseudo-first-order kinetic model was used in the following form:

$$Ln\frac{C_{t}}{C_{o}}=-Kt$$

where *C_o_* and *C_t_* are the DBT concentrations (mg/L) at equilibrium and at respective time *t*, and *K*_1_ is the pseudo-first-order rate constant that was calculated from the intercept of the plot of *Ln.C_t_/C_o_* versus time (*t*). Figure 6a,b shows the pseudo-first-order and pseudo-second-order kinetic models, respectively, for catalytic ODS of DBT in the presence of MnO_2_/MrGO catalysts, whereas the kinetic parameters are given in Table 1. As evident from Figure 6a, a nonlinear plot was obtained for pseudo-first-order kinetic model, having an R^2^ value of less than 0.99, which indicates that the catalytic ODS of the modeled oil sample does not follow pseudo-first-order kinetics. The oxidative conversion of DBT was also interpreted by the pseudo-second-order kinetic model, which is given by Equation (2),

(2) $$\frac{t}{q_{t}}=\frac{1}{k_{2}q^{2}}+\frac{1}{q_{e}t}$$

A plot of *t/q_t_* against time (*t*) was constructed for DBT conversion which gives the linear line (Figure 6b) with an R^2^ value of 0.99. The value of q_exp._ is also very close to q_calc._, indicating that catalytic ODS of DBT follows pseudo-second-order kinetics. The values of pseudo-first-order and pseudo-second-order rate constants and other kinetic parameters for DBT conversion are listed in Table 1, which represent the reactivity of the sulfur compounds model in the presence of MnO_2_/MrGO catalyst. 

### 3.4. Regeneration and Recycling of the Catalyst 

**Figure 7:**
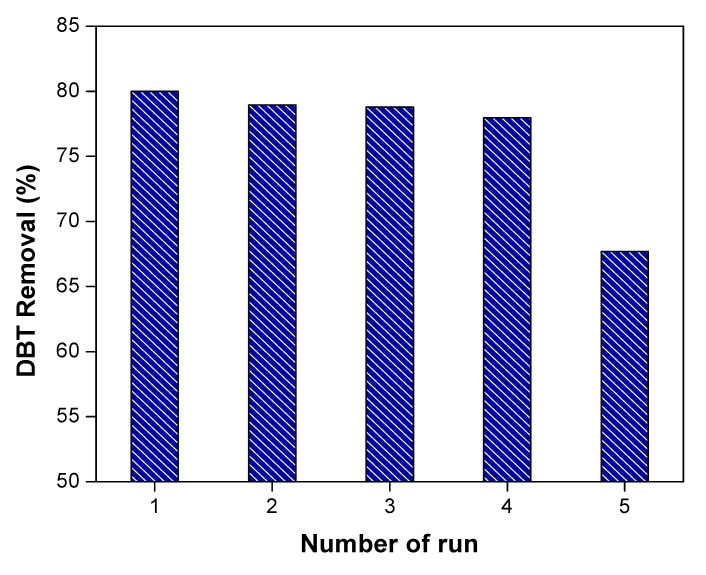
The effect of catalyst recycling (1–5 runs) on DBT conversion. Conditions: 15 mL of 1200 ppm DBT solution, 2 mL H_2_O_2_/HCOOH, 0.08 g catalyst, and 15 min reaction time at 40 °C.

Catalyst recycling is crucial for controlling the processing cost in terms of practical implementation. Regeneration of MnO_2_/MrGO was assessed by carrying out five multiple ODS experiments under similar optimized conditions. Upon the completion of each experiment, the catalyst was recovered by an external magnetic field, washed with n-heptane and methanol several times to remove leftovers of any DBT or DBT-sulfones from the catalyst’s surface. The catalyst was dried in an oven at 110 °C and then reused in another batch experiment. The result shows (Figure 7) that DBT conversion efficiency remained almost the same after four consecutive cycles but then abruptly fell. The loss of sulfur removal efficiency up to 11% after the fifth cycle was comparable with some earlier reports [15], which were not adopted in the current study, taking into account cost and safety.

The large drop in the catalyst efficiency for the fifth cycle may be attributed to a decrease in the availability of active sites on the catalyst surface. The DBT or DBTO_2_ formed during the ODS process may be adsorbed on catalyst surface at the active sites, i.e., MnO_2_ through π-complexation [48], which cannot be removed by simply washing with methanol and heptane during the regeneration step. In each cycle, a slight decrease in active sites occurs, which consequently results in a large drop in efficiency for the fifth cycle. 

### 3.5. Catalytic ODS of Commercial Oil Sample 

**Figure 8:**
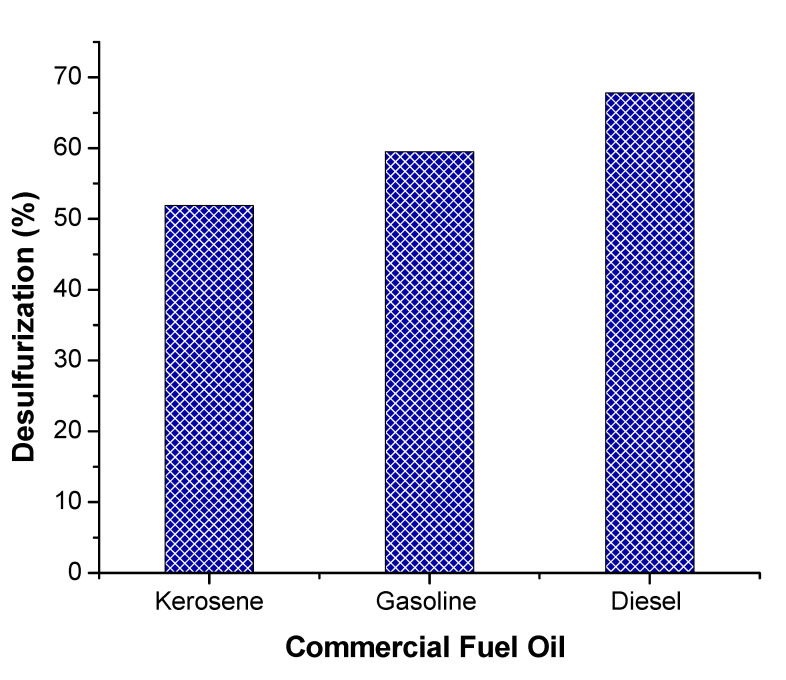
Desulfurization (%) of commercial fuel oil at 40 °C temperature and reaction time of 60 min in the presence of MnO_2_/MrGO, fuel volume (15 mL), catalyst weight (0.08 g), oxidant volume (2 mL), reaction time (15 min), and temperature (40 °C).

The catalytic ODS of commercial oil samples, including gasoline, kerosene, and diesel oil, having total sulfur contents of 3950 ppm, 6120 ppm, and 6530 ppm, respectively, was studied under optimized conditions for the modeled oil sample. Figure 8 shows that the total sulfur removal attained by catalytic ODS, in the case of gasoline, kerosene, and diesel oil, was 59%, 51%, and 67%, respectively. These results imply that the catalyst also performed well in the case of real oil samples, as in the modeled oil sample. The efficiency of the present catalytic ODS system in the case of real oil samples is comparable with those reported in the literature [44]. As reported, the ODS process, which includes the extraction step to remove sulfur content for straight run gas oil (SRGO) and diesel oil, presented high sulfur content up to 70% and 96%, respectively [49]. Another study reported on the ODS of gasoline oil, in which the sulfur content was removed up to 56.3% at 55 °C after 30 min of reaction time, followed by an extraction stage, which is a costly and time-consuming process [50].

According to the data, it is speculated that among gasoline, kerosene, and diesel oil samples, the highest sulfur removal is attained in the case of gasoline, followed by diesel oil and kerosene oil. The highest desulfurization yield is also attained in the case of diesel oil by MnO_2_/MrGO catalysts. This is possibly due to its high sulfur content because more sulfur-containing active sites of the catalysts are available to interact compared to the cases of kerosene or gasoline [51]. Similarly, the high level of sulfur removal in the case of gasoline is explained based on the nature of sulfur compounds. Mostly in gasoline, sulfur compounds are simpler and more susceptible to oxidation compared to those contained in high boiling point fractions [52]. However, in the case of kerosene oil, not only the sulfur content is low but also the prevailing sulfur compounds are more complex, implying high resistance to oxidation and therefore lower level of sulfur removal [53]. The efficiency of the current catalytic ODS system surpasses those reported for ODS of modeled and real oil samples in terms of cost-effectiveness, simplified operation, and utilization of H_2_O_2_/HCOOH as oxidant.

### 3.6. Suggested Mechanism for ODS of DBT by MnO_2_/MrGO

**Figure 9:**
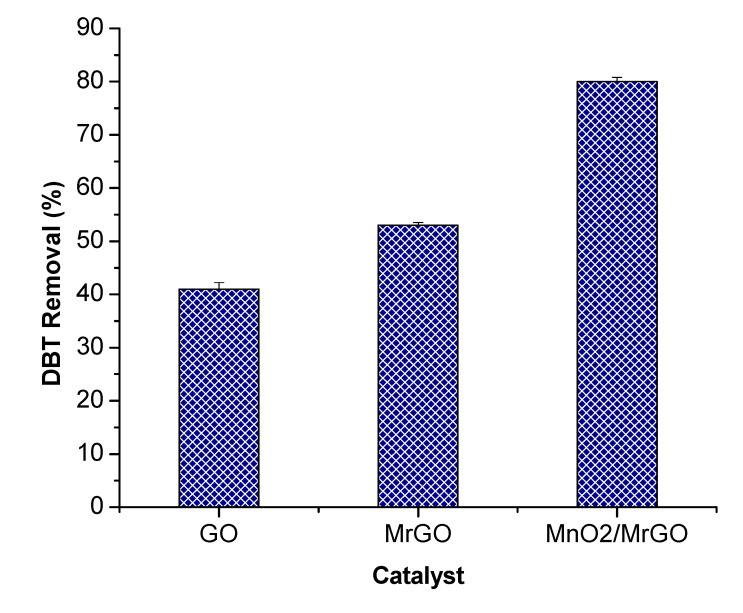
ODS of the modeled oil sample in the presence of GO, MrGO, and MnO_2_/MrGO_._

In order to confirm the catalytic role of MnO_2_/MrGO composite, ODS of the modeled oil sample was examined separately in the presence of GO, MrGO, and MnO_2_/MrGO composite as catalysts using H_2_O_2_/HCOOH as the oxidation system. In Figure 9, it is apparent that in the presence GO, MrGO, and MnO_2_/MrGO, DBT conversion was found to be 41%, 53%, and 80%, respectively. These results show that the ODS activity of GO is very small; moreover, the incorporation of magnetic iron oxide causes only a slight increase (about 12%) in the ODS activity of GO. In contrast, the ODS activity was enhanced to 80% with the incorporation of MnO_2_. Therefore, it may be suggested that the GO and the magnetic iron oxide may contribute to the DBT removal through the adsorption process [54]; however, the active component in the catalyst is MnO_2_, which causes a marked increase in the ODS activity of the composite catalyst. 

The advantage of the composite catalysts containing both MrGO and MnO_2_ is evident because DBT removal is sufficiently increased compared to only GO as the catalyst. Several studies showed that metal oxides loaded on GO and other such supports (i.e., zeolites or alumina) increase the desulfurization yield in the presence of different oxidation systems [55,56]. In ODS reaction, these metal oxides lead to the formation of peroxo species, which can oxidize sulfur compounds. For example, using MoO_x_/Al_2_O_3_ as a catalyst with H_2_O_2_ as the oxidizing agent, DBT oxidation takes place through the formation of hydroperoxymolybdate species, which were formed upon the electrophilic attack of H_2_O_2_ over octamolybdate and heptamolybdate species [57]. In the current system, manganese dioxide supported on MrGO is assumed to enhance the oxidation of DBT, leading to higher desulfurization yield than GO because MnO_2_/MrGO offered high DBT removal efficiency. 

**Scheme 1:**
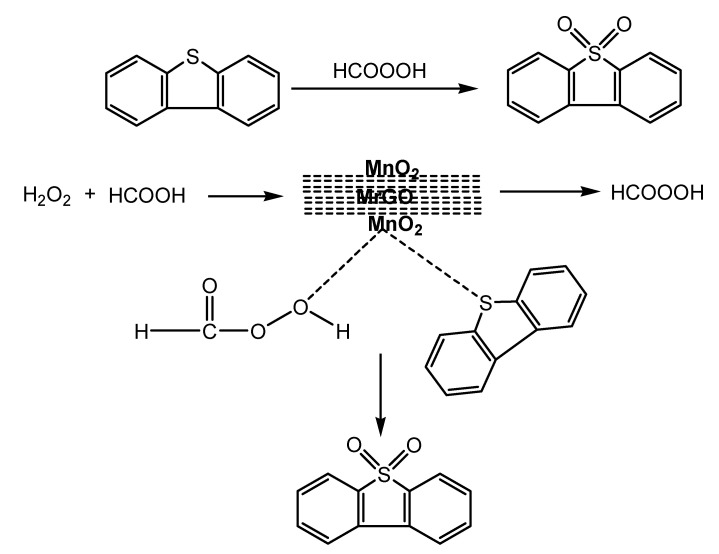
Mechanism of ODS in the presence of MnO_2_/MrGO_._

An appropriate pathway is presented for a better understanding of the mechanism of ODS by the H_2_O_2_/formic acid system in the presence of MnO_2_/MrGO as the catalyst. In the current study, the reaction is initiated by MnO_2_, involving the heterolytic cleavage of H_2_O_2_, and thus producing active hydroxyl radical (OH**^.^**); hydroxyl radicals are strong oxidizing agents [40], which further attack formic acid to produce performic acid. Performic acid offers its oxygens to DBT in order to form DBT sulfoxide and then DBT sulfone. It is also possible that the peroxyl group, produced by the reaction between H_2_O_2_ and HCOOH, interacts with the surface of MnO_2_, which carries out the selective oxidation of the S atom when DBT is adsorbed on the catalyst’s surface [58]. The proposed mechanism is presented in Scheme 1.

Here, GO acts as support, but due to its two-dimensional geometry, electron transfer capability, and ability to form π-complexes with sulfur compounds, it further enhances the desulfurization yield [59]. 

The comparison between the efficiency of the current catalytic ODS processes using MnO_2_/MrGO catalyst and those reported in the literature are provided in Table 2. Various catalytic ODS processes that removed sulfur from modeled and real oil samples using different types of catalysts through the ODS process operated at longer reaction times and temperatures. In the current study, the ODS of modeled and commercial oil samples was operated at mild operating conditions, i.e., 60 min at 40 °C with high desulfurization yield, which is comparable to the reported ones. The current process exhibits high sulfur removal efficiency in a relatively shorter duration and lower temperature. In addition, the current process does not require an extraction step, which makes it economically feasible and a less time-consuming process. These results have suggested that the combination of MnO_2_ and GO could be an excellent support offering enhanced ODS activity [34].

## 4. Conclusions

To summarize, the ODS of modeled and commercial oil samples was investigated using a MnO_2_/MrGO composite as the catalyst. The higher desulfurization efficiency attained was up to 80% in 15 min at 40 °C, using 0.08 g of MnO_2_/MrGO as the catalyst. The catalytic ODS was found to follow the pseudo-second-order kinetic model. MnO_2_/MrGO catalyst realized high desulfurization efficiency for the fuel oil sample and decreased the sulfur content up to 67.8%, 59.5%, and 51.9% in cases of diesel, gasoline, and kerosene oil, respectively. The current study accredited that ease of operation, low cost, availability of raw materials, operation at mild conditions, and high efficiency can be envisioned for fuel processing on an industrial level.

## Figures and Tables

**Table 1:** Kinetic parameters for ODS of model oil in the presence of MnO_2_/MrGO catalysts.

Order of Reaction	q_e_ (Experimental)	q_e_ (Calculated)	K (min^−1^)	R^2^
Pseudo-first-order	326.54	247.15	0.029	0.75
Pseudo-second-order	334.44	357	0.0028	0.99

**Table 2:** A comparative analysis of the performance of ODS processes containing various catalysts and supports.

Catalysts	Oxidant Used	Substrate	Temp	Time	Desulfurization (%)	Ref.
Polyoxometalate K_6_ [α-P_2_W_18_O_62_]·14H_2_O and K_6_P_2_W_18_O_62_/GO	Octanal/air	DBT, BT and 4,6-DMDBT	60 °C	-	96.10	[60]
HPW-GO	H_2_O_2_	DBT, BT and 4,6-DMDBT	60 °C	30 min	100%	[34]
MPc/RGO	O_2_	DBT	60 °C	180 min	97.5%	[61]
CuW/TiO_2_–GO	H_2_O_2_	Gasoline oil	40 °C	1 h	100%	[62]
SBA-15-supported peroxophospho-tungstate catalysts	H_2_O_2_	DBT, Gasoline	70 °C	2 h	98%, 89%	[63]
GO/COOH	H_2_O_2_/HCOOH	DBT	25 °C	300 min	95%	[64]
Molybdenum anchored/MOF	TBHP	DBT, diesel fuels	70 °C	14.4 h^−1^	85%, 74%	[65]
Methyltrioxorhenium in ionic liquid	H_2_O_2_	Thiophene, gasoline	60 °C	2 h	99%, 91%	[66]
Activated carbon	H_2_O_2_/HCOOH,	DBT, diesel fuels	60 °C	1 h	98%	[67]
K6P2W18O62/GO,	Air/n-octanol	DBT	60 °C	2 h	92.99%	[60]
MnO_2_/MrGO	H_2_O_2_/HCOOH	DBT Real oil samples	40 °C	15 min	80%	Current study

## Data Availability

Data sharing not applicable, all the data created for this study is already displayed in the article.

## Abbreviations

GO	Graphene oxide
rGO	reduced Graphene oxide
MrGO	Magnetic reduce graphene oxide
TBHP	Tertiary butyl hydroperoxide
SRGO	Straight run gas oil
MMT	Montmorillonite
RON	Research Octane Number
